# High School Students’ Preferences and Design Recommendations for a Mobile Phone–Based Intervention to Improve Psychological Well-Being: Mixed Methods Study

**DOI:** 10.2196/17044

**Published:** 2020-07-09

**Authors:** Ulrika Müssener, Kristin Thomas, Preben Bendtsen, Marcus Bendtsen

**Affiliations:** 1 Department of Medical and Health Sciences Linköping University Linköping Sweden

**Keywords:** mental health, stress, high school students, intervention, mHealth

## Abstract

**Background:**

Young adults’ mental health is characterized by relatively high rates of stress and anxiety and low levels of help-seeking behavior. Mobile health (mHealth) interventions could offer a cost-effective and readily available avenue to provide personalized support to young adults. More research needs to be directed at the development of mHealth interventions targeting youths specifically, as well as at determining how to reach young people and how to effectively intervene to improve psychological well-being.

**Objective:**

The objective was to gather perceptions from high school students to inform the development of a prototype mHealth intervention aiming to promote psychological well-being.

**Methods:**

A mixed methods design was used to (1) investigate high school students’ perceptions about stress and its consequences in daily life, as well as their ability to cope with stress, and (2) explore their preferences and design recommendations for an mHealth intervention to improve psychological well-being. Students from two high schools in the southeast of Sweden were invited to take part in the study. Recruitment of high school students was completed over a 6-week period, between October 25 and December 7, 2018. Recruitment entailed inviting students to complete a stress test (ie, screening and feedback) on their mobile phones. After completing the stress test, all participants were invited to complete a follow-up questionnaire and take part in telephone interviews.

**Results:**

A total of 149 high school students completed the stress test, of which 68 completed the questionnaire. There were 67 free-text comments distributed across the items. The majority of participants (55/68, 81%) stated that they coped with stress better or in the same way after engaging in the stress test, due to time management, dialogue with others, and self-refection. A total of 4 out of 68 participants (6%)—3 female students (75%) and 1 male student (25%)—took part in telephone interviews. Three main themes were identified from the interview data: perceptions about stress, design features, and intervention features.

**Conclusions:**

Stress was described by the students as a condition caused by high demands set by oneself and the social environment that impacted their physical health, personal relationships, school performance, and emotional well-being. Participants claimed that mHealth interventions need to be clearly tailored to a young age group, be evidence based, and offer varied types of support, such as information about stress, exercises to help organize tasks, self-assessment, coping tools, and recommendations of other useful websites, literature, blogs, self-help books, or role models. Mobile phones seemed to be a feasible and acceptable platform for the delivery of an intervention.

## Introduction

### Mental Health Among Youths and the Educational Sector as a Setting for Efforts

Mental health disorders account for about half of the disease burden among young adults in developed countries [1] and threaten health outcomes and academic performance [2-5]. Perceived high demands, peer relationships, academic pressure, lack of time management, parental pressure, school environment, recreational activities, low global self-esteem, and low social support have been identified as crucial factors that explain mental health problems in adolescents (eg, stress symptoms, sleeping problems, or worry and anxiety) [2,3].

Adolescents are in a vulnerable phase during which their future health behavior is being shaped. Behaviors developed during this period influence health in adulthood [1,6]. Most young people experiencing mental health problems do not access medical or professional services, leading to a risk to develop mental health disease later in life (eg, depression). Stigma and embarrassment about seeking help, confidentiality concerns, and poor mental health literacy are barriers to help-seeking [3,5,6].

The education sector can play an important role in mental health promotion among youths, especially when it comes to promoting stress management [7]. The schools’ multidisciplinary teams provide good access to adolescents and school is a natural setting for mental health promotion [8]; as well, the context is appropriate because the stressors and demands on students partly concern school-related issues [9].

So-called psychosocial educational interventions, aiming to promote mental health, have been implemented in school settings. These interventions can be provided in meetings between the students, as individuals or in groups, and the teacher, mentor, or health professionals at the school health center [10,11]. These interventions include techniques to improve students’ psychosocial health, relaxation training, stress management, problem solving, mindfulness, social adjustment, and emotional self-control. However, such interventions have demonstrated a limited effect on reducing symptoms of stress [12] and in preventing depression in the short term [11]. Also, psychosocial educational interventions are time-consuming, and school staff are not able to fully address these important issues due to high workloads and limited resources [3].

### Mobile Health Interventions to Improve Psychological Well-Being

To address both the need to scale up interventions and to reach the groups that tend to be reluctant to seek help, and to offer efforts that require minimal resources and time, interest has increased to provide interventions via mobile phones. Such interventions fall under the umbrella term mobile health (mHealth) interventions, defined as a medical or public health practice that is supported by mobile devices [13]. mHealth interventions could offer a cost-effective and readily available avenue to provide personalized support to young adults. This intervention medium is especially suited for this group for several reasons. Mobile phone ownership and use are nearly universal. In addition, mobile phone interventions can be utilized at little cost and can be delivered at any time, to any location, with high fidelity. These interventions can also be utilized without the stigma of having to inform friends, family members, or school staff that one is seeking mental health support [14].

A recent meta-analysis showed that mHealth interventions can promote mental health, including stress management, among university students [15]. However, systematic reviews and meta-analyses [16-19] of the use of mobile apps and text messaging for mental health issues revealed several important limitations. These included lack of control or comparison groups, small sample sizes, an absence of theory-based interventions, and a high attrition rate. In addition, mHealth interventions are not sufficiently explained theoretically, not analyzed in enough detail to determine mechanism of change, and often focus on specific patient groups [20].

A fast-growing area of research is the documentation and critical analysis of the formative research process required in the development and refinement of effective mHealth interventions [21]. Systematic reviews underscore the urgent need for examining development processes for mHealth interventions [22] in order to fully understand how interventions have been developed to allow replication and adaptation of interventions across settings [23]. Previous research has pointed out that the most important factors during the design process is to be flexible and responsive to the input and feedback of the target audience: if they do not enjoy the program they may disengage [24]. More research needs to be directed at the development of mHealth interventions targeting youths specifically, as well as at determining how to reach young people and how to effectively intervene to improve psychological well-being.

### Objectives

In this study, we aim to gather perceptions from high school students to inform the development of a prototype mHealth intervention aiming to promote psychological well-being.

## Methods

### Study Design

A mixed methods design was used to (1) investigate high school students’ perceptions about stress and its consequences in daily life, as well as their ability to cope with stress, and (2) explore their preferences and design recommendations for an mHealth intervention to improve psychological well-being. Data collection was conducted through questionnaires and individual interviews. The study design followed recommended standards for mHealth development [24,25] and explored both preferences among the target audience regarding an mHealth intervention and general perceptions and experiences of stress.

### Recruitment

Students from two high schools, aged between 15 and 19 years, in the southeast of Sweden were invited to take part in the study; the student population consisted of about 1000 students. The inclusion criteria for taking part in the study were as follows: (1) able to read and understand Swedish, (2) willing to complete a digital stress intervention (ie, screening and feedback), and (3) own a mobile phone. The research project was advertised at the schools through printed media (ie, posters and leaflets), digital media (ie, student email and school website), and verbal information delivered by school staff in the classrooms.

Recruitment of high school students was completed over a 6-week period, between October 25 and December 7, 2018. Recruitment entailed inviting students to complete a stress test (ie, screening and feedback) on their mobile phones. The stress test was previously developed by our research group and consists of screening of stress symptomatology using the Perceived Stress Scale [26] and one item investigating sleep quality during the past month. The Perceived Stress Scale is a validated scale that measures global stress [26]. The stress test encompassed screening and feedback. The test gives feedback on stress levels and recommends stress-management strategies based on screening results. These strategies are all common evidence-based strategies, such as progressive muscle relaxation as well as cognitive behavioral therapy–based and mindfulness-based stress-reduction techniques.

Students registered their interest by sending a text message to a dedicated telephone number; they immediately received a confirmation message with information about the study and a link to the stress test. A total of 14 days after completing the stress test, all participants were invited, via text message, to complete a follow-up questionnaire and take part in telephone interviews.

### Materials and Data Collection

The follow-up questionnaire aimed to investigate students’ abilities to cope with stress, perceptions of the feedback given, and perceptions about support to cope with stress. The questionnaire included six items that had been generated for the purpose of this study (see Multimedia Appendix 1).

The aim of the interviews was to explore students’ perceptions about stress and its consequences in daily life. Informants were also asked about their experiences of the stress test and perceptions about future mHealth interventions targeting stress among high school students. A semistructured interview guide was used [27]. The interview questions were framed around the following domains: (1) experiences of stress and its consequences on daily living, (2) use of the mobile phone for health informatics, and (3) user requirements and implementation aspects for an mHealth intervention to reduce stress. The interviewer was the first author of this manuscript (UM), a female researcher with a PhD degree and training and experience in qualitative methodology. The interviews lasted 45-60 minutes. Participants expressed their interest in taking part in the interview by leaving their names and phone numbers at the end of the questionnaire. The interviews were performed between February 17 and March 15, 2019.

### Data Analysis

#### Questionnaire Data

Descriptive analysis [28] was used for the distribution of the responses to the questionnaire data. Free-text items were analyzed using manual content analysis [29]. First, all free-text data were read by the first author. Second, the free-text data were discussed among the authors, and comments that captured the main content of each specific question were selected. The free-text data were used to underline and illustrate the pattern of response to the fixed-response options.

#### Interview Data

Thematic analysis was used to analyze the professionally transcribed interview data [27]. The analyses followed a prescribed, sequential process: overall impressions were noted, data were reduced and coded into initial themes, we searched for patterns and interconnections, final themes were mapped and built, and conclusions were drawn [27]. The first author read the transcriptions to acquire a comprehensive understanding. The selected quotes were then compared between the authors, and agreement was reached on the quotes to be included. Statements that were not clearly in line with the purpose of this study were excluded. Themes were identified using an iterative process of reading and rereading the transcripts. Patterns were searched for, and coding into themes was initiated by the first author. Next, the themes were presented and discussed among all authors, and boundaries for themes were established jointly. Excerpts from the interview transcripts are presented to support and exemplify the categorization. The interviewees’ fictitious names are given after their quotes, followed by gender and age.

### Ethical Considerations

The study was approved by the Regional Ethical Committee in Linköping, Sweden (Dnr: 2018/269-31).

## Results

### Quantitative Data From the Stress Test

#### Overview

A total of 149 high school students—107 female and 42 male students—completed the stress test and were, therefore, invited to complete the questionnaire; 68 participants, all between 16 and 19 years old, completed the questionnaire. Overall, there were 67 free-text comments distributed across the items. Most comments related to perceived benefits of the intervention (n=18).

#### Ability to Manage Stress

The majority of the participants (55/68, 81%) stated that they coped with stress better or in the same way after engaging in the stress test, 2 participants (3%) stated that they handled stress worse after than before, and 11 (16%) stated that they did not know. Data showed that participants perceived stress differently and had learned how to prioritize their time:

I remember to think about the tips in certain situations I notice that I start to get stressed [about] and perhaps choose to say “No” to something I didn’t have to do so that I get to do something else, which lowers my stress.

I have noticed that I have started to see stress from a different perspective. If, for example, I am anxious about something and start stressing, I think, for example, that this will also pass.

In contrast, data showed that learning about stress and raising participants’ awareness could elevate stress levels:

I experience that I become more aware of stress and feel stressed about being stressed.

A total of 12 free-text answers concerned the reasons why participants handled stress better now than before the intervention (eg, time management, dialogue with others, and self-refection); a few representative quotes are shown:

I believe it’s probably because I try to plan my time better.

That I think more about what makes me stressed. I find it easier to let go of things. I have tried to follow some of your advice. It has worked well and calmed me down; it has also helped me to minimize problems that I previously had blown up out of proportion, specifically because I feel life is easier when I stop and think about how I feel.

I have talked with others and thought more about how I handle stress. I therefore think that I unconsciously handle stress in a better way.

#### Perceptions About the Feedback Given

A total of 40 participants out of 68 (59%) found the feedback—tips, advice, and exercises—given to reduce stress to be *Good* or *Very good*. Among those who left free-text comments for this question, some stated that being screened for stress symptoms led to an awakening and increased awareness; they perceived not only that they were not as stressed as they thought, but also that they needed to manage stress differently:

It was like an “Aha” moment that I am too stressed, when I got a number put on it, because I have always thought that it was normal and that everybody else was just as stressed as me, but the number meant that I have started to think about it, and that I should learn how to handle my stress better.

It made me realize that I do not stress as much as I think I do. That I can categorize my stress as necessary and unnecessary.

However, only 14 participants out of 68 (21%) claimed that they had benefitted from the stress test. The participants underlined the need for tips and exercises to reduce stress in everyday life but pointed out the difficulty in following the given tips and asked for more tangible advice:

They were excellent tips, but [I] think the problem is actually putting those nine tips into practice.

The feedback was good, but to have an effect perhaps we need more concrete tips.

A total of 54 participants out of 68 (79%) answered *No* or *Don’t know* to the question “Did you find the feedback usable?” Suggestions for improvements concerned the need to be able to revisit the feedback:

For me, the exercises have worked well; however, I thought it was a great shame that you couldn’t go back and see the exercises and advice again after the test was finished. That is something that could definitely be improved.

I did not have too much time to do them or test them.

Furthermore, participants reported that the intervention content (ie, tips and advice) were redundant, well-known, and already available on the internet. Similarly, specific expert feedback was requested:

[I] didn’t get any tips that I didn’t already know about or used.

These are comments that you can easily search for on the internet. [I] would have liked more tips from a professional who is an expert on all of this.

#### Additional Support

Out of 68 participants, 30 (44%) indicated that they would recommend the test to a friend and 12 (18%) would not. A total of 14 (21%) participants contributed with suggestions for additional support. The following suggestions are examples given in the free-text comments:

1. “Planning and how to prioritize your work.”
2. “Perhaps a video clip where you can listen to somebody who has had a problem and has solved it.”
3. “I am less stressed after being physically active if it is a type of exercise I enjoy.”
4. “Reminders on your mobile [phone] to destress.”
5. “A hobby is very good, like painting, dancing, singing, etc.”

### Qualitative Data From the Interviews

#### Overview

A total of 4 participants out of 68 (6%)—3 female students (75%) and 1 male student (25%)—took part in telephone interviews. Three main themes were identified in the data: (1) perceptions of stress, (2) design features, and (3) intervention features (see Textbox 1).

**Three main themes and their subthemes identified from the thematic analysis.**

Perceptions about stress:

- Experiences
- Consequences

Design features:

- Content characteristics
- Stress education
- Coping strategies

Reaching young adults:

- Duration and timing of support
- Enrollment
- Delivery

#### Perceptions About Stress

Perceptions about stress included both experiences and perceived consequences of stress.

#### Experiences

Participants described stress as a condition caused by high demands set by oneself, the school, and the social environment. High expectations of coping with stress in multiple settings emerged as a prominent trigger of actual stress and something that further contributed to a perceived imbalance in life:

But the type of stress I experience is very much linked to school, friends, and all the demands around this, the context, such as you must be slim, you must get good grades, you must exercise a lot, go to lots of parties, and, you know, all the pieces, that everything falls into place...I feel demands from my family that I have to behave in a certain way; I must be nice, help with the food, washing, and chores.

Carl, 16 years, male

School, schoolwork, trying to balance life with school, get good grades. It’s not possible.

Greta, 16 years, female

Experiences of stress were also described as something positive, forcing one to get things done:

For me, stress is like both good and bad, when you know that there is something that must be handed in soon, and then you get stressed and so you do the things that have to be done; then it is a good thing and you actually do the things you have to.

Carl, 16 years, male

#### Consequences

Consequences of stress included feelings of frustration, being overwhelmed, and being unproductive when unable to prioritize. Furthermore, participants expressed that stress impacted their physical health, personal relationships, school performance, and emotional well-being. For example, students described health issues, such as migraine and sleep difficulties. Others explained that their school performance suffered due to the loss of concentration and not being able to prioritize. Regarding personal relationships, participants described difficulties remaining connected with friends:

When I have a lot to do and when I don’t know what to prioritize, then I feel I get stressed. When I don’t know what to start on first. And then it can end up with me not doing any of it; that can be the consequence of it...[I] often get quite frustrated when I don’t know how to tackle it. So I have problems focusing on one thing.

Maria, 17 years, female

You can become so stressed that you just shut down, you can’t manage any more, you’re just too stressed, you can’t even think...Sometimes I got so stressed that I got a migraine.

Greta, 16 years, female

I have had an awful lot of difficulty sleeping...You just carry the crap inside you and do not have the energy to keep up contacts with everybody.

Carl, 16 years, male

#### Design Features

#### Overview

Participants described what they thought would be important and valuable components of an mHealth intervention targeting mental health among high school students. Design features could be divided into three subthemes: content characteristics, stress education, and coping strategies.

#### Content Characteristics

Characteristics that the participants highlighted included that the content should be clearly tailored to a young age group, be evidence based, and offer varied types of support. For example, one participant stressed the importance of involving high school students in intervention development and creating a product that young people could relate to:

It’s good that you keep in contact with, like, young people who, like, calls and talks with them, really have young people involved who keep track of this process, because I can promise you that if you want to have good contact with young people and reach them, having their opinions will really help.

Greta, 16 years, female

Furthermore, the value of interventions being evidence based was emphasized in the interviews; for instance, participants expressed that interventions and guidance grounded in research and best practice increase users’ confidence to participate. Further, the need for recommended dedicated literature was also expressed as important to include in future interventions. References to other literature and support by an expert competent in the specific field of knowledge would help to screen the large volume of information available:

I listen a lot to things that are scientifically proven. I would think it was great to hear facts...you know, what is important and what you should listen to...it would be fantastic if somebody, an expert, who knows about these things could make recommendations.

Carl, 16 years, male

The importance of providing varied support to reach the many different personalities and needs was further highlighted by participants:

That it should be easily explained, and then that it should work for many people, as many as possible. That it isn’t just text messages, but that perhaps, yes, but one could vary it a lot, I think that would be good.

Maria, 17 years, female

I think that you must be able to provide many different things in it, but that you have selected out that which is good.

Susanne, 17 years, female

When asked about the preferred style and an appropriate tone for the text messages, the clear response was that text should not be overly positive or optimistic:

Don’t just be super positive and optimistic!...“Oh—keep fighting, after the rain comes the sun”; nobody wants to hear this, I can tell you...because if I get that sort of message, I just, like, delete that contact and block them, I don’t want to hear that. Many, many, many of us are like that.

Greta, 16 years, female

#### Stress Education

Providing information about stress appeared to be fundamental. Participants stated that being provided with relevant information would help them to cope with stress and anxiety. Some described the need for access to information on how stress manifests and how to normalize stress. They also emphasized the importance of having a deeper understanding of their own reactions as a coping tool:

I think get knowledge about how it works, how it can affect you, at least for me I have got a good understanding and I have been able to manage it better. But then I’ve got help, that I now know how it works and what you can do about it.

Maria, 17 years, female

That there are different ways of looking at stress, so that you don’t get stuck in your own bubble that “Oh, I have so much to do now,” because I think I get even more stressed from that now, because one should get, I don’t know what it could be, but something that gets you to open up a little and understand a little more that “Yes, but OK, it isn’t only me that is stressed.”

Susanne, 17 years, female

Following their need for information, participants suggested the inclusion of self-assessment in a future intervention to estimate their level of stress and to get to know their own stress triggers. Participants welcomed messages that included a link to a self-assessment survey and that gave regular feedback on their stress levels. They believe that such content could support self-reflection and make it easier to navigate when searching for the most useful support:

Exactly this with a test, that can also be good...just to be able to self-assess. Because then you reflect over yourself...it is important to know where you lie, that you get, so that you get the right type of relaxation and support.

Carl, 16 years, male

Participants stressed their need for recommendations of other useful websites, literature, blogs, self-help books, or role models. Some proposed that the intervention could have an accessible platform where they could choose various types of support from a range of resources with recommendations to relevant literature. The data showed that teenagers want quick results and consequently request shortcuts to useful support:

Honestly, I think you can send out recommendations to pages, recommendations to other things that you know are good, books...links to self-help books and other websites or blogs or such things. No, but you know, all teenagers want fast results so they can get on or something or be better at things quickly.

Greta, 16 years, female

Using people who had been in a similar situation and who had benefited from similar support as role models was suggested as a recommendation for other forms of support:

Yes, but perhaps it could present people who have been stressed but have perhaps gone through a similar program and feel less stressed. That you have a role model sort of. That it works so long as you don’t stop. And that it isn’t the same all the time, you have to have something new that isn’t complicated.

Carl, 16 years, male

#### Coping Strategies

Time constraints and an inability to meet targets and organize time schedules were burning issues that triggered students’ stress. All participants underlined the need for support to structure and organize tasks that had to be done at school and in daily living. They wanted advice and exercises to help organize tasks. Regarding the difficulties in structuring and prioritizing tasks, participants requested support in practicing how to take on one thing at a time. Furthermore, reminders or notifications to take time out and to do something else to relax were proposed:

Well I would like someone who says “Yes, but do this task now, then after you have done this task, then we move on to the next one.” Like someone who, I know organizes it, or what one should say, very clearly with “Everything you are to do now is important, but you must start with one task so that you move forward,” because I feel that I don’t do that, I get stuck in what I have to do first...one needs to slow down a little so that you can test these exercises and see if you can remember how you should think and things like that.

Maria, 17 years, female

Definitely advice. So that you do something that breaks what you are stressed about and it doesn’t spin out of control.

Greta, 16 years, female

Like, OK to be stressed now, but you can do something about it and then you can do this like, yes, but a little bit. Have a support, like.

Maria, 17 years, female

Some participants suggested recommendations for relaxation exercises and meditation. Others claimed that any kind of activity that decreases stress would be useful and proposed that a future intervention should encourage taking up a hobby or leisure activity:

What I really like is meditation, for relaxing, if you come home from school or have problems sleeping, putting on a meditation video and just listening to it, that is fantastic to do...important with advice on how to relax, anything...and just a reminder to take two seconds for yourself.

Greta, 16 years, female

Yes, but like get a hobby so you get away from it all for a little while, so to speak...but some people, they, like, want to go out and run, some want to sit and paint, some want to do yoga, find something.

Carl, 16 years, male

#### Reaching Young Adults

##### Overview

Participants described ways and preferences on how the target group could be reached and supported in their stress management. The data included suggestions on the methods for recruitment and delivery that would optimize a future mHealth intervention.

##### Duration and Timing of Support

The data showed that preferences regarding the length of a proposed intervention, the number of text messages sent per week, and the timing of messages differ from person to person:

It depends on the person. But a couple of months. I think, that for it to have an effect that it would have to be for more than a month...I wouldn’t appreciate getting it every day. Because then I think I would feel a little overwhelmed. That isn’t how you want it. Not continuously all the time. That would be too much. Then you’d just ignore it. But perhaps every third day.

Carl, 16 years, male

I think, like, every other day, because you don’t want to get too much information about it or you wouldn’t want it to feel forced, or I would at least...Then I think perhaps three weeks or thereabouts, three perhaps four.

Maria, 17 years, female

Participants indicated that simple messages, such as reminders sent a couple of times per week, with the aim of encouraging them to take a break, might be helpful in creating a sense of being supported by someone who cares about their situation:

But like, perhaps twice a week or something, not often but just a reminder every so often.

Greta, 16 years, female

The importance of being able to make one’s own decision regarding the amount of support and duration of the intervention was stressed in the interviews:

So perhaps, say that you can have different types, if you can sign up say for a month or two months or whatever it is...Yeah, but I think that is best, because then it also gives you a feeling of having more control, which everybody wants to have, they want to be in control, because nobody wants [to] be completely out of control, without feeling “Yes, but I can choose whether I will have more or less.”

Greta, 16 years, female

Concerning the timing of the delivery of the text messages, participants suggested delivery during the afternoon or evening:

And at the times perhaps when people are most stressed, I don’t really know when that is, but perhaps some time in the afternoon when you get home from school and have to cram. Or in the evening, in the evening perhaps it would be good to get a reminder that you should go to bed early...not in lectures and such and on breaks when you have other things to do, I think, than listen to a program; you have to focus on what needs to be done.

Susanne, 17 years, female

##### Enrollment

The data showed that preferences regarding recruitment channels varied, indicating that high school students are a heterogeneous group and that employing multiple recruitment strategies is optimal. Strategies such as school staff advertising with posters in the assembly rooms and meeting halls, digital advertising through emails, and information in the classrooms were described as successful. Furthermore, social media was highlighted as a recruitment channel (eg, Instagram or Snapchat):

The teachers mean a lot, and things that they are behind, you listen to...If our teachers talk with us, we have mentoring every Monday, for example, then we sit and listen to what they are saying and they, that was when this test thing came up, the whole class did it. I think via the notice board in the school is best, because if we see it on Instagram, it is easy to just, once again, to just scroll down or ignore it. The school will always be a very good way to reach young people about things like this.

Greta, 16 years, female

That is a good way [via the school], but I think perhaps it could be good in some way to show oneself on social media. Instagram and Snapchat. But, as I said, the school is also a good way to go.

Carl, 16 years, male

##### Delivery

Participants found mobile phones to be a feasible and acceptable platform for the delivery of an intervention. Text messages, especially, seemed to be an appreciated method:

Well I thought it was a great idea that you get it directly to your mobile phone...I think it would be nice with SMS because otherwise it is easier to avoid doing anything about it, choosing not to go in and open the app and things.

Maria, 17 years, female

## Discussion

### Principal Results

This mixed methods study investigated how high school students perceive stress and their preferences regarding support for stress management. Our aim was to increase our knowledge in the area to develop an mHealth intervention aiming to promote psychological well-being among high school students. The questionnaire data showed that the stress test format (ie, screening and feedback) offered good stress-management support and increased the awareness about stress among participants. However, the data also showed that future interventions would benefit from more tangible advice and the ability for users to revisit advice and tips.

Three main themes were found in the interview data: perceptions about stress, design features, and intervention features. In general, the qualitative data showed that this population is a fairly heterogeneous group with varied support preferences and needs (eg, in regard to the timing and duration of an intervention). Also, the data showed that high demands from various life areas contribute to stress and that this group expects quick, easily accessible support and guidance on how to cope with stress. Finally, interview data suggested that mobile phones are useful as a platform for stress management but that specific features need to be considered for optimal reach.

### Comparison With Prior Work

In this study, stress was described as a condition caused by high demands set by oneself and the social environment (eg, by the school and by people significant to the students). The most prominent cause of stress was high expectations of coping with stress in multiple settings. Previous research has stressed that young persons are actors in a social context in which they interact with, for example, peers, technology, social norms, and body ideals. Research points out that these cultural and social contexts need to be considered if we are to understand and approach mental health among young adults [30]. Furthermore, our data indicated that stress impacted on various life areas, such as physical health, personal relationships, school performance, and emotional well-being. Our findings highlighted the interest and curiosity among high school students regarding evidence-based information on how stress manifests and how to normalize stress among young adults. Following the need for information, some participants suggested the inclusion of self-assessment in a future intervention to estimate their level of stress. However, recent research problematizes the picture of youth mental health and points out the difficulty of measuring students’ well-being in general, and stress in particular, based on symptom frequency.

A growing discussion in society concerns a need to distinguish in-depth problems from those of a more everyday nature. There is currently a major emphasis on diagnosis: a goal to provide a medical diagnosis and subsequently offer treatment [3,31]. For young people suffering from mental health problems but who are not “ill enough,” there are no easy inroads to receiving scientifically reliable help for problems such as anxiety, sleeping difficulties, feeling down, and stress-related psychosomatic problems [3]. Thus, there is a need for future research to develop interventions targeting subclinical populations.

The findings from this study highlight the potential difficulty in accommodating a fairly heterogenous group with mHealth interventions. Although the challenge of tailoring intervention content and structure is relevant for any mHealth intervention, certain elements may be specific for the young adult age group. Our findings showed that high school students value specific features, such as evidence-based content that is aesthetically pleasing and easy to navigate. Furthermore, the findings showed that this group expects quick, easily accessible support, and they down-prioritize support that does not meet these criteria. Considering the vast number of mHealth interventions that are commercially available, this group could be challenging to reach if interventions are not specifically tailored to their age group and accommodating to these aspects [6,13,17,22,32].

High school students seemed to have a great amount of trust in the school staff, and they recommended recruitment via school health services. The results confirm the declaration made from the World Health Organization that the education sector can play an important role in health promotion among youths [13]. Furthermore, participants found mobile phones to be a feasible and acceptable platform for the delivery of an intervention. Text messages, especially, seemed to be an appreciated way to deliver the intervention, which is in line with the growing body of evidence supporting the significance of mHealth interventions to help people modify health behaviors [14].

### Strengths and Limitations

This study has several limitations. First, a relatively short questionnaire was used to explore the views of the participants. Second, the interviews were few (n=4). As in all qualitative studies, the sample size was not based on power calculations, but rather on data saturation. When and how saturation is reached varies from study design to study design; hence, there is no one-size-fits-all method to reach data saturation. However, according to previous research using qualitative design [27], one way to think of data is in terms of rich and thick, rather than the size of the sample; that is, to differentiate between rich and thick data is to think of *rich* as high quality and *thick* as high quantity. One can have a lot of thick data that is not rich; conversely, one can have rich data but not a lot of it. In this study, we chose to conduct the research in a manner allowing us to reach data saturation by collecting rich (ie, high-quality) and thick (ie, high-quantity) data. We account for multiple sources of data and perspectives to ensure that the study results demonstrate validity through data saturation. Hence, strengths in this study were the use of a mixed methods design; analyses of the questionnaire data were enhanced when the quantitative data were viewed in relation to the patterns found in the qualitative interview data. Findings not captured or understood through numeric answers alone were exposed through the variations in data. Several steps have been taken to ensure the validity of the results. Regarding the questionnaire data, two authors read the free-text comments independently many times. The first author selected a variety of the most crucial free-text comments, and then the chosen free-text comments were presented and discussed with the other authors; comments that captured the main content of the specific question regarding the aim of the study were chosen. Free-text comments that were not agreed on by all authors were excluded. A similar procedure was taken when analyzing the interview data; two authors independently coded and analyzed the transcripts to improve the trustworthiness of the findings.

### Conclusions

The adolescent period is characterized by rapid physical and psychological changes in the individual, together with increasing demands and influence from peers, school, and wider society. Including young people’s own perspectives is of relevance to psychosocial health. Youths’ experiences of feeling psychosocially well or not need to be heard and collected as basic information in scientific studies, including in the development of new interventions. This mixed methods study demonstrated the advantages of conducting a formative appraisal early in the process to guide the development of a prototype mHealth intervention aiming to promote psychological well-being. These findings will become imperative when designing a forthcoming mHealth intervention based on high school students’ needs, perspectives, and recommendations.

The findings can contribute to a deepened understanding on how high school students experience stress and on important aspects of future stress-management support targeting this population. Indeed, the findings were helpful in learning about our intended target group, in assessing the health issue, in starting to identify the components of possible contents, in exploring the natural settings, and in how to reach young adults.

## Appendix

Multimedia Appendix 1 Follow-up questionnaire.

## Abbreviations

mHealth: mobile health

## References

[1] (2013). Mental Health Action Plan 2013-2020.

[2] Schraml K, Perski A, Grossi G, Makower I (2012). Chronic stress and its consequences on subsequent academic achievement among adolescents. J Educ Dev Psychol.

[3] Ahrén JC (2010). Skolan och Ungdomars Psykosociala Hälsa [School and Adolescents’ Psychosocial Health]. SOU 2010:80.

[4] Hagquist C (2010). Discrepant trends in mental health complaints among younger and older adolescents in Sweden: An analysis of WHO data 1985-2005. J Adolesc Health.

[5] Lillevoll KR, Vangberg HCB, Griffiths KM, Waterloo K, Eisemann MR (2014). Uptake and adherence of a self-directed internet-based mental health intervention with tailored e-mail reminders in senior high schools in Norway. BMC Psychiatry.

[6] Mason M, Ola B, Zaharakis N, Zhang J (2015). Text messaging interventions for adolescent and young adult substance use: A meta-analysis. Prev Sci.

[7] Haraldsson KS, Lindgren EM, Fridlund BG, Baigi AM, Lydell MC, Marklund BR (2008). Evaluation of a school-based health promotion programme for adolescents aged 12-15 years with focus on well-being related to stress. Public Health.

[8] Kraag G, Zeegers MP, Kok G, Hosman C, Abu-Saad HH (2006). School programs targeting stress management in children and adolescents: A meta-analysis. J Sch Psychol.

[9] de Anda D, Baroni S, Boskin L, Buchwald L, Morgan J, Ow J, Gold JS, Weiss R (2000). Stress, stressors and coping among high school students. Child Youth Serv Rev.

[10] SBU (2010). Methods to Prevent Mental Ill-Health in Children: A Systematic Review.

[11] Merry S, Hetrick SE, Cox GR, Brudevold-Iversen T, Bir JJ, McDowell H (2011). Psychological and educational interventions for preventing depression in children and adolescents. Cochrane Database Syst Rev.

[12] Kraag G, Zeegers MP, Kok G, Hosman C, Abu-Saad HH (2006). School programs targeting stress management in children and adolescents: A meta-analysis. J Sch Psychol.

[13] WHO Global Observatory for eHealth (2011). mHealth: New Horizons for Health Through Mobile Technologies. Second Global Survey on eHealth.

[14] Mason M, Ola B, Zaharakis N, Zhang J (2015). Text messaging interventions for adolescent and young adult substance use: A meta-analysis. Prev Sci.

[15] Conley CS, Durlak JA, Shapiro JB, Kirsch AC, Zahniser E (2016). A meta-analysis of the impact of universal and indicated preventive technology-delivered interventions for higher education students. Prev Sci.

[16] Gliddon E, Barnes SJ, Murray G, Michalak EE (2017). Online and mobile technologies for self-management in bipolar disorder: A systematic review. Psychiatr Rehabil J.

[17] Rathbone AL, Prescott J (2017). The use of mobile apps and SMS messaging as physical and mental health interventions: Systematic review. J Med Internet Res.

[18] Versluis A, Verkuil B, Spinhoven P, van der Ploeg MM, Brosschot JF (2016). Changing mental health and positive psychological well-being using ecological momentary interventions: A systematic review and meta-analysis. J Med Internet Res.

[19] Donker T, Petrie K, Proudfoot J, Clarke J, Birch M, Christensen H (2013). Smartphones for smarter delivery of mental health programs: A systematic review. J Med Internet Res.

[20] Riley W, Rivera DE, Atienza AA, Nilsen W, Allison SM, Mermelstein R (2011). Health behavior models in the age of mobile interventions: Are our theories up to the task?. Transl Behav Med.

[21] Maar MA, Yeates K, Toth Z, Barron M, Boesch L, Hua-Stewart D, Liu P, Perkins N, Sleeth J, Wabano MJ, Williamson P, Tobe SW (2016). Unpacking the black box: A formative research approach to the development of theory-driven, evidence-based, and culturally safe text messages in mobile health interventions. JMIR Mhealth Uhealth.

[22] Badawy SM, Kuhns LM (2017). Texting and mobile phone app interventions for improving adherence to preventive behavior in adolescents: A systematic review. JMIR Mhealth Uhealth.

[23] Ricci-Cabello I, Bobrow K, Islam SMS, Chow CK, Maddison R, Whittaker R, Farmer AJ (2019). Examining development processes for text messaging interventions to prevent cardiovascular disease: Systematic literature review. JMIR Mhealth Uhealth.

[24] Abroms LC, Whittaker R, Free C, Mendel Van Alstyne J, Schindler-Ruwisch JM (2015). Developing and pretesting a text messaging program for health behavior change: Recommended steps. JMIR Mhealth Uhealth.

[25] US Department of Health and Human Services, National Institutes of Health, National Cancer Institute (2002). Making Health Communication Programs Work: A Planner's Guide.

[26] Cohen S, Kamarck T, Mermelstein R (1983). A global measure of perceived stress. J Health Soc Behav.

[27] Patton MQ (2015). Qualitative Research & Evaluation Methods: Integrating Theory and Practice. 4th edition.

[28] Leavy P (2017). Research Design: Quantitative, Qualitative, Mixed Methods, Arts-Based, and Community-Based Participatory Research Approaches.

[29] Miles MB, Huberman AM (1994). Qualitative Data Analysis: An Expanded Sourcebook. 2nd edition.

[30] Wickström A, Zetterqvist Nelson K, Johansson T, Sorbring E (2018). Psykisk hälsa ur barns och ungdomars perspektiv: De sociala relationernas avgörande betydelse [Psychological health from children’s and young people’s perspectives: The meaning of social relationships]. Barn- och Ungdomsvetenskap: Grundläggande Perspektiv [Child and Adolescent Science: Basic Perspectives].

[31] Schraml K, Perski A, Grossi G, Simonsson-Sarnecki M (2011). Stress symptoms among adolescents: The role of subjective psychosocial conditions, lifestyle, and self-esteem. J Adolesc.

[32] Hetrick S, Cox GR, Witt KG, Bir JJ, Merry SN (2016). Cognitive behavioural therapy (CBT), third-wave CBT and interpersonal therapy (IPT) based interventions for preventing depression in children and adolescents. Cochrane Database Syst Rev.

