# Supplementary material for: High School Students’ Preferences and Design Recommendations for a Mobile Phone–Based Intervention to Improve Psychological Well-Being: Mixed Methods Study
Source: JMIR Pediatr Parent. 2020 Jul 9;3(2):e17044. doi: 10.2196/17044 (PMC7381011; doi:10.2196/17044)
Supplement: Multimedia Appendix 1 [file pediatrics_v3i2e17044_app1.docx]

Appendix 1. Questionnaire.

1. Has your ability to manage stress changed since you completed the test and were offered tips, advice and exercises? (Response options: I handle stress worse now than before/I handle stress in the same was as before/I handle stress better now/I don’t know).

2. If you manage stress better now, what are the reasons? (only free text comment).

3. You received feedback on your perceived stress level. What did you think of the feedback? Please leave suggestions for improvement and/or other comments. (Response options: Very good/Good/Neither good nor bad/Very bad/Don’t know).

4. The feedback you were given included tips, advice and suggestions for exercises to reduce your stress. Did you benefit from these tips, advice and exercises? Please leave comments on your experiences and/or suggestions for improvement (Response options: Yes/No/Don’t know).

5. Do you have suggestions for other support useful for reducing your stress? Please describe what kind of tips, advice and exercises you would prefer (Response options: Yes/No/Don’t know).

6. Would you recommend the test to a friend? (Response options: Yes/Not sure/No/Don’t know).
